# Brain effects of 5:2 intermittent fasting and the healthy living diet in a randomized controlled trial

**DOI:** 10.1002/alz.084536

**Published:** 2025-01-09

**Authors:** Apostolos Manolopoulos, Roger J Mullins, Francheska Delgado‐Peraza, Maja Mustapic, Carlos Nogueras‐Ortiz, Pamela J. Yao, Mark R Cookson, Josephine M. Egan, Sophia Frangou, Mark P. Mattson, Dimitrios Kapogiannis

**Affiliations:** ^1^ National Institute on Aging/National Institutes of Health, Baltimore, MD USA; ^2^ Morgan State University, Baltimore, MD USA; ^3^ Cell Biology and Gene Expression Section, Laboratory of Neurogenetics, National Institute on Aging, National Institutes of Health, Bethesda, MD USA; ^4^ National Institute on Aging, Baltimore, MD USA; ^5^ Mt. Sinai School of Medicine, New York, NY USA; ^6^ Center for Brain Health University of British Columbia, Vancouver, BC Canada; ^7^ Johns Hopkins University School of Medicine, Baltimore, MD USA

## Abstract

**Background:**

Insulin Resistance (IR) is implicated in brain aging and Alzheimer’s disease (AD) pathogenesis. Dietary changes may promote brain health in older adults with metabolic abnormalities. An extensive animal literature suggests pro‐cognitive and beneficial systemic and brain effects of intermittent fasting (IF) that may mitigate AD risk. We conducted a randomized clinical trial comparing brain effects and the potential for AD biomarker modulation of IF and a continuous diet.

**Method:**

Forty overweight, cognitively intact individuals > 55 years old with peripheral IR were randomized (1:1) to 5:2 IF (2 days 480 Kcal/day; 5 non‐restricted days) or Healthy Living (HL) diet by USDA recommendations for 8 weeks.

**Result:**

Both IF and HL improved biomarkers of brain IR in neuron‐derived extracellular vesicles (NDEVs), decreased regional BrainAGE (brain‐age‐gap estimate on structural MRI) in the anterior cingulate and ventromedial prefrontal cortex, reduced glucose concentration on brain magnetic resonance spectroscopy (MRS) indicating optimized metabolism, and improved executive function and memory. Moreover, both diets decreased weight, BMI, and waist circumference suggesting high compliance, and HOMA2‐IR indicating IR alleviation. They also increased blood beta‐hydroxybutyrate and acetoacetate suggesting increased ketogenesis. Although effects of IF and HL were statistically comparable, greater numerical improvements were observed with IF for several measures of brain health, cognition, and peripheral metabolism. However, cerebrospinal fluid (CSF) and NDEVs showed no changes in AD biomarkers (Aβ_42_, total Tau, p181‐Tau, GFAP). In exploratory analysis, effects were moderated by polymorphisms in ApoE ε4 genotype, with cognitive benefits and decreased MRS glucose solely in ε4 non‐carriers; ε4 carriers showed no cognitive benefits, no decreases in MRS glucose, but increased CSF Aβ_42_/Aβ_40_ ratio, NfL and GFAP.

**Conclusion:**

Beneficial effects of HL and, especially, IF on NDEV‐derived brain IR, BrainAGE, brain glucose concentration, executive function and memory indicate that healthy diets may improve brain health even over short periods of time, albeit with no evidence for AD cascade modulation. Genetic factors, such as ApoE ε4 polymorphisms, may influence the brain’s response to diets. Future clinical trials encompassing IF and other dietary interventions and stratified by ApoE ε4 carrier status may provide avenues for slowing the pace of brain aging.

